# Visual loss secondary to eosinophilic mucin rhinosinusitis in a woman: a case report

**DOI:** 10.1186/1752-1947-4-350

**Published:** 2010-10-29

**Authors:** Anurag Garg, Raja Das-Bhaumik, Alex D Nesbitt, Adam P Levene, Naresh Joshi, William E Grant, Angus Kennedy

**Affiliations:** 1 Department of Neurology, Chelsea and Westminster Hospital, London SW10 9NH, UK; 2 Department of Ophthalmology, Chelsea and Westminster Hospital, London SW10 9NH, UK; 3 Department of Histopathology, St Mary's Hospital, London W2 1NY, UK; 4 Department of ENT, Charing Cross Hospital, London W6 8LH, UK

## Abstract

**Introduction:**

Eosinophilic mucin rhinosinusitis is an inflammatory pathological condition of the nose and paranasal sinuses. It is rare, occurs in immunocompetent patients and is characterised by peripheral eosinophilia and extensive bilateral sinus disease. To the best of our knowledge, visual loss with this condition has not been previously reported.

**Case presentation:**

We present the case of a 26-year-old Asian woman with a background history of chronic sinusitis who presented with acute left-sided visual loss. Imaging showed significant opacification in the frontal, ethmoidal and sphenoidal sinuses as well as evidence of a unilateral optic neuritis. Histological analysis of sinus mucin revealed dense eosinophilic infiltrate and, despite medical and surgical intervention, vision was not restored in her left eye.

**Conclusion:**

We introduce visual loss as a complication of eosinophilic mucin rhinosinusitis. This adds further evidence to previous reports in the literature that optic neuropathy in sinusitis can occur secondary to non-compressive mechanisms. We also describe a rare finding: the vision in this patient did not improve following steroid therapy, antifungal therapy or surgical intervention. There are very few such cases described in the literature. We conclude that chronic sinusitis is an indolent inflammatory process which can cause visual loss and we reiterate the importance of recognizing and considering sinusitis as a cause of visual loss in patients in order that prompt medical and surgical treatment of the underlying disease can be initiated.

## Introduction

Eosinophilic mucin rhinosinusitis (EMRS) is an inflammatory pathological condition of the nose and paranasal sinuses. It is a rare type of chronic sinusitis which is thought to occur secondary to systemic eosinophilic dysregulation [1]. Clinically, it is characterised by peripheral eosinophilia and extensive bilateral sinus disease. It typically occurs in immunocompetent individuals who frequently also have asthma, nasal polyposis and a previous history of sinus surgery. Pathologic findings of EMRS include abundant eosinophilic infiltrate and debris but no demonstrable fungal hyphae [1,2].

To the best of our knowledge, ophthalmic manifestations of EMRS have not previously been described in the literature. We present the case of a young woman with a background history of chronic sinusitis who presented with acute left-sided visual loss. Imaging showed significant opacification in the frontal, ethmoidal and sphenoidal sinuses as well as evidence of a unilateral optic neuritis. The histological analysis of the sinus mucin revealed dense eosinophilic infiltrate and, despite medical and surgical intervention, vision was not restored in the left eye. We conclude that EMRS can cause visual loss and reaffirm that chronic sinusitis is an important underlying cause that should be considered in any patient presenting with a loss of vision.

## Case presentation

A 26-year-old Asian woman presented with a four day history of unilateral left-sided altitudinal visual loss associated with painful eye movements, headaches, nasal obstructive and catarrhal symptoms. She was asthmatic and had undergone endoscopic sinus surgery and nasal polypectomy for chronic sinusitis five months previously.

On more detailed questioning, she described a three day history of gradual loss of sight occurring from the superior to the inferior aspect of her vision - 'like a shadow' falling over her left eye where she was able to see 'grey only' in the upper half of her left visual field with the lower half appearing 'blurry'. In addition, she had been concurrently experiencing dull pain around her left eye and on eye movements, especially left eye adduction.

Over the next 24 hours her vision further deteriorated. She was now able to see 'grey only' in the whole left visual field at which point she presented to hospital. She had been suffering from a background of nasal congestive symptoms and intermittent headaches for the previous ten days.

On admission, her visual acuity was 6/4 in her right eye and limited to perception of light in her left eye in all quadrants. In her left eye, there was a relative afferent pupillary defect and red desaturation. Eye movements were normal. Fundoscopy of her left eye revealed optic disc swelling but nothing else; the macula was normal, there was no vascular sheathing and spontaneous venous pulsation was present.

**Figure 1:**
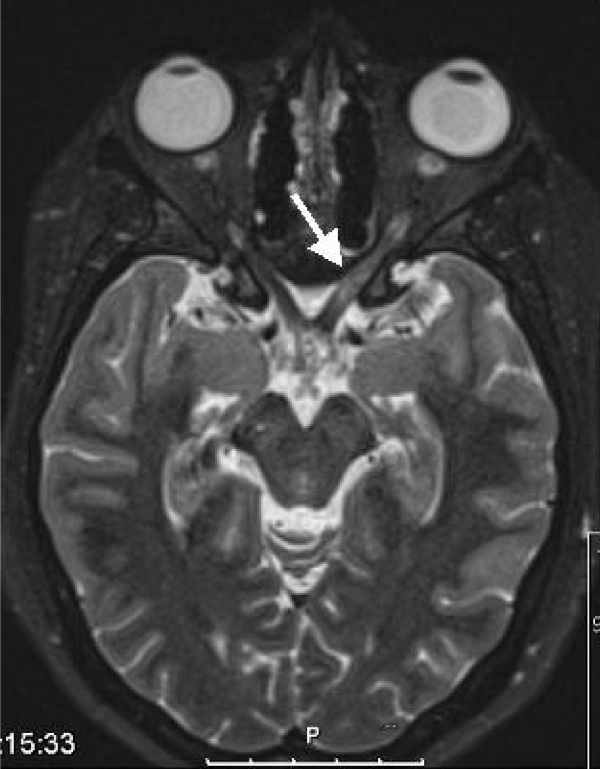
**T2 weighted magnetic resonance imaging (MRI) image of the orbits**. Nine days after the onset of visual loss, MRI shows slight swelling of the left optic nerve just proximal to the chiasm with mild signal changes also demonstrated.

Computed tomography of the brain showed normal intracranial appearances but opacfication of frontal, ethmoidal and sphenoidal sinuses. Magnetic resonance imaging showed an increased signal in the left optic nerve proximal to the optic chiasm suggestive of neuritis but no evidence of optic nerve compression (Figure 1).

Blood tests revealed mild peripheral eosinophilia (absolute eosinophils = 0.8 × 10^9^/L, normal interval: 0.0-0.4 × 10^9^/L) though overall white cell count normal (8.8 × 10^9^/L) and other white cell differential count unremarkable (absolute lymphocytes = 2.5 × 10^9^/L, normal interval: 1-3.5 × 10^9^/L; absolute monocytes = 0.4 × 10^9^/L, normal interval: 0.3-1 × 10^9^/L; absolute neutrophils = 5.1 × 10^9^/L, normal interval: 2-7.5 × 10^9^/L; absolute basophils = 0.1 × 10^9^/L, normal interval: 0-0.1 × 10^9^/L).

Inflammatory markers showed slightly elevated ESR (14 mm/hour) and normal C-reactive protein (7 mg/L). Serum IgM 2.26 g/L (normal interval: 0.50-1.90 g/L) was raised though other antibodies were within normal ranges: serum IgG 14.2 g/L (normal interval: 5.4-16.1 g/L); serum immunoglobulin A 2.29 (normal interval: 0.8-2.80 g/L); and serum immunoglobulin E 99 kU/L. Other laboratory findings included: haemoglobin 13.4 g/dL, platelet count 378 × 10^9^L, normal liver function and renal function, HIV status negative, syphilis serology negative and lyme serology negative.

A lumbar puncture was performed which revealed a normal opening pressure (11 mmHg). Cerebrospinal fluid (CSF) protein electrophoresis showed no evidence of immunoglobulin G oligoclonal bands. CSF direct microscopy/culture showed no organisms on Gram stain and no growth at two days.

Visual evoked potential testing showed absent P100 cortical responses to full field monocular stimulation of her left eye using both large and small check sizes consistent with a left optic neuropathy. The right eye studies were within the normal latency limits.

**Figure 2:**
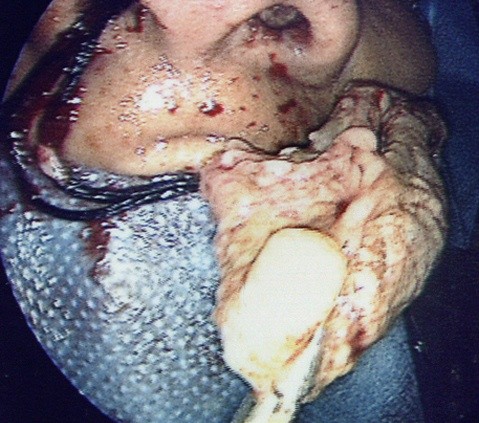
**Photograph of the thick 'axle grease' mucin removed from sinuses**.

She was treated with intravenous augmentin, amphotericin and methylprednisolone and four days later underwent radical sphenoethmoid disease clearance, revealing thick 'axle-grease' mucin (Figure 2).

A sphenoethmoidectomy was completed to the level of the skull base, with wide sphenoidotomies and antrostomies fashioned. After the disease clearance, the walls of the sphenoid sinuses were inspected but no bony defect was found. The lamina papyracea were inspected on both sides but no defect was found.

**Figure 3:**
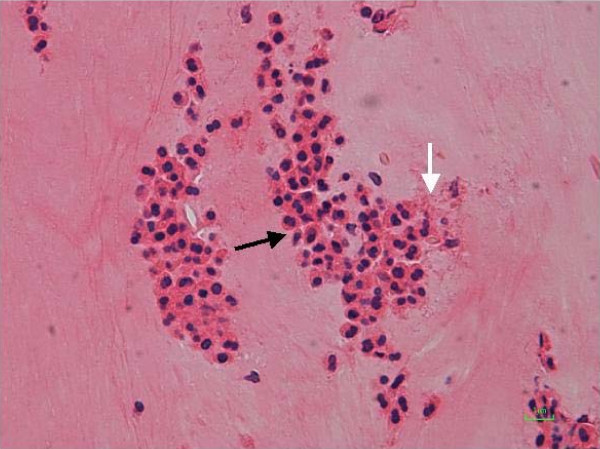
**Haematoxylin and eosin stain (×40)**. Layers of degenerating eosinophils (solid arrow) with abundant eosinophilic debris (empty arrow).

A histological analysis of the mucin and polypoid inflammatory tissue revealed abundant eosinophilic infiltrate and eosinophilic debris but no demonstrable fungal hyphae (Figure 3). Fungal cultures were negative. A diagnosis of EMRS was made.

She was discharged ten days later on oral voriconazole and prednisolone. Visual acuity was to hand movements in her left eye and 6/4 in the right. At one month, there was gradual improvement to counting fingers in her left eye.

## Discussion

EMRS is a form of chronic rhinosinusitis which was first described after clinicopathological differences were noted between patients with allergic fungal sinusitis (AFS) and a subset of patients demonstrating characteristics of AFS but in whom fungal cultures or stains were negative.

Ferguson described other differences between the two groups, noting the fungal culture/stain negative cohort had an overall older age of disease onset, exclusive bilateral sinus disease distribution and a very strong association with asthma [1].

Although AFS represented a localised type 1 hypersensitivity reaction to fungal agents, EMRS was a systemic immunological disease occurring secondary to systemic eosinophilic dysregulation. Both conditions typically occurred in immunocompetent patients who also frequently had nasal polyposis and a previous history of sinus surgery.

In this, the patient had a background history of asthma, nasal polyposis and chronic rhinosinusitis. Mucin samples taken following operative disease clearance were sent for fungal culture and histological analysis revealed no fungal growth and no demonstrable hyphae, although histology demonstrated abundant eosinophilic infiltrate and eosinophilic debris.

These features strongly suggest a diagnosis of EMRS and, using the AFS diagnostic criteria [3], she did not exhibit features of a type 1 hypersensitivity response.

In a recent review of the literature, Aakalu *et al. *determined that 33 patients have been reported to have had partial or complete visual loss from AFS [4]. It had been proposed that visual loss in these cases were a result of different mechanisms including mechanical compression of the optic nerve [5] (directly or indirectly), secondary to orbital inflammatory changes causing an optic neuritis [6-8], venous congestion of the optic nerve due to thrombophlebitis and retinal artery occlusion due to increased orbital pressure [9]. Treatment has been centered primarily on surgical decompression of the optic nerve [5], though steroid therapy [6] has also shown benefit.

To the best of our knowledge, there have been no reports of cases of visual loss occurring secondary to EMRS. In addition, previous reports of visual impairment related to AFS have shown dramatic improvement in visual acuity following treatment with optic nerve decompression, steroid therapy and fungal immunotherapy [4]. However, in this case, despite all three interventions, our patient's visual acuity did not improve significantly.

The pathogenesis remains unclear. In the absence of compression, possible mechanisms would include a reactive optic neuritis secondary to adjacent inflammatory sinus disease, similar to cases reported to have AFS. It is also possible that a reactive vasculitis caused an ischemic neuropathy given the altitudinal field defect.

## Conclusion

We present the case of a young immunocompetent woman who presented with acute visual loss due to EMRS. This unusual case highlights that chronic sinusitis is an indolent inflammatory process that can cause visual loss. It reaffirms the importance of considering and recognizing chronic sinusitis as a cause of visual loss and the need for the prompt initiation of medical and surgical treatment of the underlying disease.

## Abbreviations

AFS: allergic fungal sinusitis; CSF: cerebrospinal fluid; EMRS: eosinophilic mucin rhinosinusitis.

## Competing interests

The authors declare that they have no competing interests

## Authors' contributions

AG and ADN were part of the neurology team who were in charge of the patient's care. AK was the consultant neurologist in charge of the patient's care. RDB was a major contributor to the manuscript and, along with NJ, was part of the ophthalmology team who assessed the patient's visual symptoms. WEG was the ear, nose and throat surgeon who performed the surgical intervention on the patient and advised on the writing of the manuscript. APL performed a histological analysis of the mucin samples obtained from the patient's sinuses. All authors read and approved the final manuscript.

## Consent

Written informed consent was obtained from the patient for publication of this case report and any accompanying images. A copy of the written consent is available for review by the Editor-in-Chief of this journal.
